# Impact of Alcohol Outlet Density on Reported Cases of Child Maltreatment in Japan: Fixed Effects Analysis

**DOI:** 10.3389/fpubh.2019.00265

**Published:** 2019-10-04

**Authors:** Yuna Koyama, Takeo Fujiwara

**Affiliations:** Department of Global Health Promotion, Tokyo Medical and Dental University, Tokyo, Japan

**Keywords:** child maltreatment, child abuse, neglect, alcohol outlets, fixed effects analysis, panel data analysis

## Abstract

**Background:** Parental drinking habits or binge drinking are a known risk factor of child maltreatment. Though drinking habits are affected by alcohol outlet density, the direct association between alcohol outlet density and child maltreatment is still controversial.

**Purpose:** This study aimed to examine the impact of off-premises alcohol outlet density on child maltreatment cases reported to Child Guidance Centers in Japan.

**Methods:** A fixed effects model was used to investigate the association between a change in off-premises alcohol outlet density and a change in child maltreatment cases in each unit. Time-series of cross-sectional ecological data collected from across Japan over 16 years (2000 to 2015) was used, and maltreatment cases were further sub-grouped by type of maltreatment (physical, sexual, psychological abuse and neglect) and by perpetrators (father, stepfather, mother, and stepmother).

**Results:** The association between alcohol outlet density and total cases of child maltreatment was not observed (coefficient = 0.98, 95% confidence interval: −6.30, 8.25). However, alcohol outlet density was shown to be positively associated with neglect (coefficient = 3.08, 95% confidence interval: 0.54, 5.62), which indicates that 1 alcohol outlet per 1,000 adults increase would lead to 3 more neglect cases per 10,000 children. Also, a negative association was observed between a change in the incidence of total child maltreatment by father and a change in alcohol outlet density (coefficient = −3.03, 95% confidence interval: −5.78, −0.28).

**Conclusion:** The findings suggest that off-premises alcohol outlet density may have a causal effect on the increasing cases of neglect and decrease in maltreatment by father in Japan.

## Introduction

The number of child maltreatment cases reported to Child Guidance Centers has been increasing in Japan and reached 122,000 in 2016 (1), a 7-fold increase compared with that of 17,000 in 2000 (1). The impact of child maltreatment includes childhood physical and mental health (2) and adulthood physical (3–9) and mental health (8, 10–15). To combat this dramatic increase of detected child maltreatment cases, it is important to elucidate the risk factors of child maltreatment so that prevention strategies can be developed.

The World Health Organization (WHO) provided several risk factors of child maltreatment based on the ecological model, such as child factors, parent or caregiver factors, relationship factors, and community or societal factors (16). Among several risk factors outlined, parental substance addiction including alcohol addiction (17, 18) in particular has received little attention in Japan, although regulation of alcohol availability may be effective to prevent child maltreatment (19–21). Because illegal drug use is not prevalent in Japan (22), and alcohol is more common (23), we believe a focus on parental alcohol consumption habits is important. Further, alcohol consumption habits can be determined by the physical environment such as alcohol outlet density, which can be associated with the increase of child maltreatment cases in Japan.

So far, the association between alcohol outlet density and child maltreatment is explained using three main theories: the availability theory (24), the routine activity theory (25–27), and the supply and demand theory (26). In availability theory, increased alcohol outlet density is associated with increased consumption and alcohol-related harms including medical harms, injuries, crime, and violence (28). The reported association between parental drinking and family dysfunction including intimate partner violence might explain poor parenting or parental violence use toward children (29–31). For example, increased outlet density was linked to increased amount of drinking for parents who have problematic alcohol drinking habits, which was reported to be associated with physical child abuse (32). Alcohol misuse was also related to depressive symptoms, leading to low social support and neglect (33). In addition, alcohol may facilitate or incite family violence by providing a socially acceptable excuse for the negative behavior such as neglect or physical abuse (30). The routine activity theory suggests that for any crime to be committed the three components are needed: offenders, suitable targets, and absence of guardians (34). As for child maltreatment, harm (child maltreatment) occurs when there is a suitable target (i.e., children), a motivated offender (i.e., drunk parents), and the absence of effective guardians (i.e., other caregivers or community) (26). For example, with rising number of restaurants or bars, parents changed their routine behavior to drinking out, which may further encourage hanging out with friends in the community (35) or with other caregivers, potentially resulting in an increase in neglect cases. In supply and demand theory, neighborhoods with high densities of alcohol outlets are often more deprived and overcrowded, and lack resources to relieve stress and to develop social networks to prevent child maltreatment. For example, social disorganization or isolation among residents due to residential characteristics including neighborhoods with higher densities of alcohol outlets was associated with higher rates of reported child maltreatment (36).

Although several studies have attempted to explain how alcohol outlet density is related to child maltreatment (25–27, 35, 37–40), the causality on whether outlet density influences the number of child maltreatment cases has not yet clarified because the findings may simply be an ecological fallacy (37). Alternatively, unmeasured confounders, such as community-level social environment and culture, may explain the association. More robust statistical analyses or well-designed studies are needed. The issue of showing the causality of the association between the number of alcohol outlets and child maltreatment can be resolved with fixed effects analysis, which adjust for unobserved, unit-specific and time-invariant confounders. To the best of our knowledge, no studies have investigated the association using fixed effects model.

In addition, the type of outlet, such as off-premises or on-premises, may have a differential association with maltreatment (25, 38, 41). That is, off-premises outlets, where people buy alcohol to take away, may be more harmful for child maltreatment than on-premises, where people drink on the spot, such as restaurants and bars (26, 39). A study in California showed no association between on-premises outlets and physical abuse and neglect, but observed a positive association between off-premises outlets and physical abuse (26). A study in New Jersey reported a positive association between on-premises outlets and neglect, and a negative association between off-premises outlets and physical abuse (39). This differential or inconsistent association by type of outlet must be due to social context, such as social capital—that is, on-premises outlets may work as a third place to promote social capital by supplying the opportunities to socialize and to strengthen connectedness, which is a protective factor for child maltreatment (42, 43). On the other hand, off-premises can be more harmful for children because parents are more likely to drink at home, thus the probability of drinking in front of children would be high. As Japan is known as a high social capital country (44), the potential impact of on-premises outlets was strong enough to produce social capital and to prevent maltreatment. Thus, we hypothesized that an increase in the number of off-premises outlets might lead to an increase in the number of child maltreatment cases by motivating parents to drink more at home or by making community more acceptable to violence. To the best of our knowledge, there is no study examining the association between off-premises outlet density and child maltreatment in Japan.

Further, there are few longitudinal studies on outlet density and child maltreatment (25, 40), which makes it difficult to infer the causal association. To elucidate the association between parental drinking behavior and child maltreatment, we investigated the association between outlet density and child maltreatment using panel data fixed effects model.

Therefore, this study examined (1) how a change in alcohol outlet density is associated with child maltreatment, and (2) how a change in alcohol outlet density is associated with child maltreatment by maltreatment types and by perpetrator types.

## Methods

Using time-series of cross-sectional ecological data, this study was designed to analyze annual data over a 16-year period (2000–2015) from 46 prefectures in Japan, except for Fukushima, which lacks 2011 data due to the Great East Japan Earthquake (i.e., 46 prefectures for 16 years, resulting in 736 prefecture-year units). There is no demarcation change during the analyzed period.

### Data

Data on alcohol outlet density was obtained from the National Tax Agency's Annual Statistics Report. Data on the number of child maltreatment cases was derived from the Ministry of Health, Labor, and Welfare's Report on Social Welfare Administration and Services. Child welfare expenses data was sourced from the “White Paper on Local Public Finance” published by the Ministry of Finance. Data on opened dental clinics was obtained from the “Survey of Medical Institutions” published by the Ministry of Health, Labor, and Welfare. Unemployment rate and financial index data were obtained from the report “Statistical Observations of Prefectures” published by the Ministry of Internal Affairs and Communications. All data except for alcohol outlet density were available in the online database of the Ministry of Internal Affairs and Communications.

### Analysis

The dependent variable was the annual rate of reports to Child Guidance Centers on child maltreatment per 10,000 children, which reflected the number of cases the Center received from several dimension entities such as police and neighbors. Although the annual rate of reports did not include whole cases, which might cause an underestimation of the actual numbers, it was generally accepted as a valid way to measure child maltreatment (27). Reports were categorized by type of maltreatment (physical abuse, sexual abuse, psychological abuse, and neglect) and by perpetrator (father, stepfather, mother, stepmother, and others). Since the number of reports whose perpetrator was categorized as “others” was relatively small and complex because it included relatives and strangers in the same category, we included only cases categorized as “father,” “stepfather,” “mother,” and “stepmother,” in the analysis.

The independent variable was the alcohol outlet density, that is, the number of licensed retailers that sell liquor per 1,000 people over the age of 20 years in a given area. Since those who sell take-away liquor in Japan need to be licensed but those who sell liquor consumed on the spot—such as owners of restaurants or bars—do not, the data acquired in this study denotes the number of off-premises outlets.

The four control variables included are as follows: child welfare expenses (total expenses on child welfare/child under 17 years old), opened dental clinics (/1,000,000 residents), unemployment rates (unemployed/working population over 15 years old) and financial index. The child welfare expense is the total expenses of child welfare per child under age 17. Opened dental clinics are the number of dental clinics opened in that year. The financial index is the average number derived from dividing the base fiscal amount of income by the base fiscal demand amount from the past 3 years. This index indicates financial power and the greater the number means the greater the financial strength. WHO showed community and societal risk factors for child maltreatment were living inequalities, high poverty rate and unemployment rate, and availability of substances (16). Among them, local economic status and unemployment rate were added as adjustment covariates due to their potential relationship with alcohol outlet density. In addition, since dentists play a significant role in maltreatment detection (45), the number of dental clinics was also employed.

Data was analyzed using multiple fixed effects models with the assumptions that, same as in regression analysis, error is unrelated to alcohol outlet density for all the 46 prefectures over a 16-year period (2000–2015), alcohol outlet density in each prefecture is independent and identically distributed, large outliers are unlikely, and unmeasured confounders were identical within the same prefecture overtime. To test whether the models had effectively accounted for independence of observations (i.e., absence of spatial autocorrelation), the residual variation was tested using Moran's I statistic (46). The spatial weighting matrix was made for first order neighbors sharing the prefectural borders. We could not reject the possibility that the errors of the model are independent and identically distributed (Moran's I statistic = 0.00, *p*-value = 0.994), so we did not further analyze with spatial modeling, i.e., alcohol outlet density in each prefecture can be assumed independent. Since fixed effects model allowed us to remove the effects of time-invariant observed and unobserved confounding variables, we could obtain the net effect of outlet density. The aforementioned control variables were included because they were time-varying confounders. The fixed effects model was conducted with generalized linear regressions for estimating the association between change in alcohol outlet density and the incidence of child maltreatment. Model 1 adjusted for unemployment rate, welfare expenditure, dental clinics, and financial index. To explore the potential mechanism under the relationship between alcohol outlet density and child maltreatment, we further adjusted with potential mediator, the divorce rate, as Model 2. All analyses were performed with STATA 15.0.

## Results

**Figure 1:**
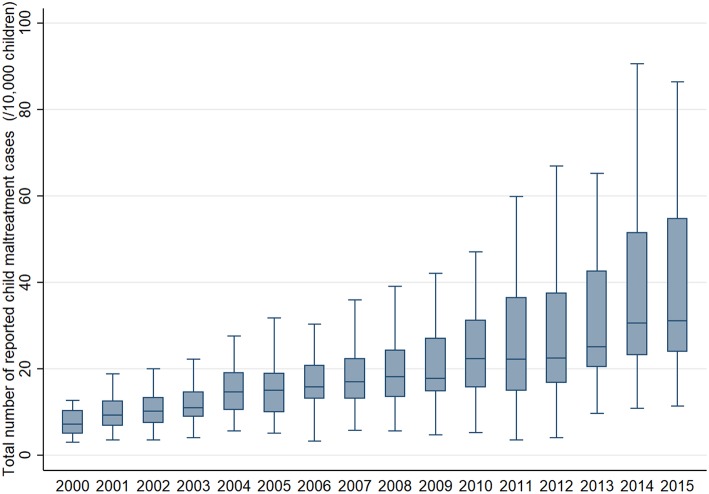
Longitudinal trend of median and variance of the total number of child maltreatment cases per 10,000 children reported to Child Guidance Center across 46 prefectures in Japan from 2000 to 2015.

The longitudinal trends of median and variance of total cases reported to Child Guidance Centers on child maltreatment per 10,000 children across 46 prefectures from 2000 to 2015 are shown in Figure 1 and those of sub-typed cases in the Supplement 1. The mean number of total cases of child maltreatment was 7.7 cases in 2000 (Standard Deviation (SD): 2.83) and 42.0 cases in 2015 (SD: 24.56). Both mean number and SD showed linear relationship with year (coefficient for trend: 2.2 and 1.2, respectively, *p*-value for trend: both <0.001), indicating the gradual increase in the total number of cases and disparities within that.

**Figure 2:**
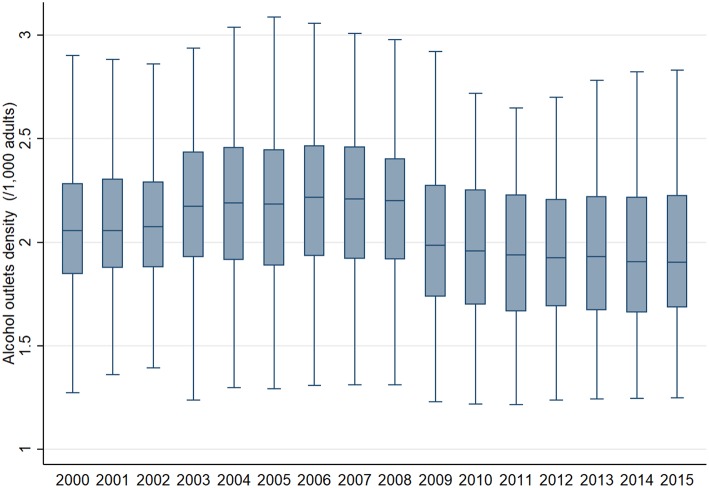
Longitudinal trend of median and variance of the number of alcohol outlets per 1,000 adults across 46 prefectures in Japan from 2000 to 2015.

The longitudinal trend of median and variance of alcohol outlet density, i.e., the number of alcohol outlets per 1,000 adults, across 46 prefectures from 2000 to 2015 is available in Figure 2. The mean number of alcohol outlet density was 2.0 in 2000 (SD: 0.34) and 1.9 in 2015 (SD: 0.34). Both the mean number and SD was almost constant over 16 years (coefficient for trend: −0.02 and −0.004 respectively, *p*-value for trend: both <0.001).

The results of fixed effects model analyses were given in Table 1. There was no association between alcohol outlet density and the total number of child maltreatment cases (coefficient (β) = 0.98, 95% confidence interval (CI): −6.30, 8.25). Seen by type, a change in alcohol outlet density was positively associated with a change in the incidence of neglect. This indicated that 3.08 (95% CI: 0.54, 5.62) neglect cases per 10,000 children under 17 would increase if 1 alcohol off-premises outlet per 1,000 people aged over 20 increases per prefecture. Sexual abuse and maltreatment by stepfather and mother showed no association (β = 0.03, 95% CI: −0.20, 0.26; β = 0.42, 95% CI: −0.95, 0.10; β = 3.05, 95% CI: −1.28, 7.37, respectively). Also, a negative association was observed between a change in the incidence of total child maltreatment by the father and a change in alcohol outlet density. Total cases of child maltreatment by father would decrease by 3.03 (95% CI: −5.78, −0.28) per 10,000 children if 1 alcohol outlet per 1,000 adults increases. Negative associations were also found for physical abuse, psychological abuse, and maltreatment by stepmother (β = −0.35, 95% CI: −2.63, 1.92; β = −1.78, 95% CI: −5.28, 1.72; β = −0.03, 95% CI: −0.17, 0.11, respectively). After further adjustment with divorce rate for the case of neglect and maltreatment by father, the number of neglect remained strongly associated with off-premise outlet density (β = 3.60, 95% CI: 1.09, 6,11) but effect size was reduced for maltreatment by father (β = −2.17, 95% CI: −4.83, 0.49).

**Table 1:** Effects of alcohol off-premises outlet density on reported cases of child maltreatment (*N* = 736).

	Model 1^*^				Model 2^**^			
	**ß**	**(SE)**	**95% CI**		**ß**	**(SE)**	**95% CI**	
Total	0.98	(3.70)	−6.30	8.25				
Physical abuse	−0.35	(1.16)	−2.63	1.92				
Sexual abuse	0.03	(0.12)	−0.20	0.26				
Psychological abuse	−1.78	(1.78)	−5.28	1.72				
Neglect	3.08	(1.29)	0.54	5.62	3.60	(1.28)	1.09	6.11
Maltreatment by father	−3.03	(1.40)	−5.78	−0.28	−2.17	(1.35)	−4.83	0.49
Maltreatment by stepfather	0.42	(0.27)	−0.95	0.10				
Maltreatment by mother	3.05	(2.20)	−1.28	7.37				
Maltreatment by stepmother	−0.03	(0.07)	−0.17	0.11				

* *Model 1 adjusted for unemployment rate, welfare expenditure, dental clinics, and financial index*.

** *Model 2 adjusted for model 1 plus divorce rate*.

## Discussion

This study explored the association between the neighborhood off-premises alcohol outlet density and rates of reported child maltreatment using fixed effects model. It revealed that the number of off-premises outlets was positively associated with the rates of neglect, and the number of off-premises outlets was negatively associated with the rates of maltreatment perpetrated by the father although it might be findings by chance or there still exists unmeasured confounders. As hypothesized, the relationship between off-premises outlets and child maltreatment differed according to maltreatment types and perpetrators, which might reveal Japanese-specific features.

The current study showed the tendency that an increase in off-premises outlets would increase child maltreatment. This finding is consistent with the previous study in California, which showed that areas with more off-premises outlets had higher rates of child maltreatment (25). As well, our study found a positive association between a change in off-premises outlets and neglect, which is inconsistent with previous studies in US that showed the positive association between on-premises outlets and neglect (26, 39). This result suggests that activity after drinking differs depending on nationality. The current study result might imply that, compared to Westerners, Japanese are less likely to attack their children after drinking too much at home, rather, they lose the interest to care for their children and end up leaving their children unsupervised. Another explanation is derived from the routine activity theory. Vulnerable drinkers might buy more alcohol if it becomes easily obtained and they tend to spend all of their money on drinking, rather than on household necessities for their children. This is consistent with another study that showed the most common reason for neglected children to be sent to protection centers was parental alcohol addiction (47).

This study also showed the negative relationship between a change in off-premises outlet density and child maltreatment by the father, which suggests that fathers were less likely to be perpetrators of child maltreatment when off-premises alcohol outlets increased in the neighborhood. Japanese fathers might be relaxed with drinking when they feel need it to avoid getting angry and acting violently because of the improved alcohol accessibility (48). In addition, our data showed significant association between divorce rates and alcohol outlet density, which implies that the potential perpetrators such as aggressive father might leave households (49). More thoroughly designed studies are needed to verify this hypothesis.

Since this study used time series data and fixed effects model analysis, which enables us to eliminate fixed time-invariant effects on the number of reports of child maltreatment resulting from prefectural characteristics, a more genuine relationship between a change in off-premises outlets and child maltreatment can be drawn. Also, our study includes data from almost all prefectures in Japan, except 1. Thus, the results of this study can be generalized to Japan.

In addition, to the best of our knowledge, this study is the first study to focus on alcohol outlet density and sexual abuse, psychological abuse, and child maltreatment by perpetrator type, as the previous studies (26, 35, 37–39) only focused on physical abuse and neglect.

Despite the above strengths, there are several limitations in this study. First, it was a prefecture-based ecological study, so the same might not be true at the individual level. It cannot be denied that these observed relationships here were the product of chance due to ecological fallacy. The spatial unit used is large enough to lead to a distortion of the effects of off-premises outlet density on the neighborhood. Also, we might miss the spillover effects that neighbor prefectures would affect individual behavior in other prefectures. However, this study used the most detailed available data. In addition, only off-premises outlets were considered in this study, which validated the definition of the unit because it is likely that people buy alcohol near their home to drink at home. Also, several studies using different units showed a similar tendency of relationship, which enabled us to infer that the relationship is not affected by the definition of study units (27). There could be, however, more robust analytical methods to consider the spatial autocorrelation such as Bayesian spatio-temporal modeling (40, 50–52). Second, since we used panel data, it was difficult to consider the duration of exposure. It is true that residents can move in and out of a given area, and environmental factors are not always the same. In order to fully examine this aspect, it is necessary to know the degree and duration of exposure to the neighborhood environment before an association with child maltreatment can be determined. Third, the actual occurrence might be underestimated in this study because only the number of child maltreatment cases reported to Child Guidance Center was counted. That is, there might be child maltreatment cases which were not reported to Child Guidance Center (53). In addition, bias toward those individuals who are more likely to be reported cannot be denied (26). A more reasonable way to count should be determined. There are several explanatory models using the availability theory, the routine activity theory, and the supply and demand theory, but there was no study that successfully showed all mechanisms of causality completely. Future studies are needed to verify the hypothesis discussed here and to discover the exact mechanism underlying the relationship between off-premises outlet density and child maltreatment.

We found no significant association between alcohol outlet density and total cases of child maltreatment. However, alcohol outlet density was shown to be positively associated with neglect, which indicates that 1 alcohol outlet per 1,000 adults induces 3 more neglect cases per 10,000 children, and negatively associated with maltreatment by father. These findings suggest that drinking habits induced by increase in off-premises outlet density may have a causal effect on increasing neglect in Japan. More thorough studies are needed to further explore the exact mechanism of off-premises outlet density and child maltreatment.

## Ethics Statement

Because we used all aggregated administrative data, no IRB approval is needed.

## Author Contributions

YK and TF conceived. YK analyzed and wrote the first draft. TF finalized the manuscript. All authors approved the final version of manuscript.

### Conflict of Interest Statement

The authors declare that the research was conducted in the absence of any commercial or financial relationships that could be construed as a potential conflict of interest.
