# Supplementary material for: Impact of Alcohol Outlet Density on Reported Cases of Child Maltreatment in Japan: Fixed Effects Analysis
Source: Front Public Health. 2019 Oct 4;7:265. doi: 10.3389/fpubh.2019.00265 (PMC6787550; doi:10.3389/fpubh.2019.00265)

Supplement 1. Longitudinal trends of the number of sub-typed child maltreatment cases reported to Child Guidance Center in Japan


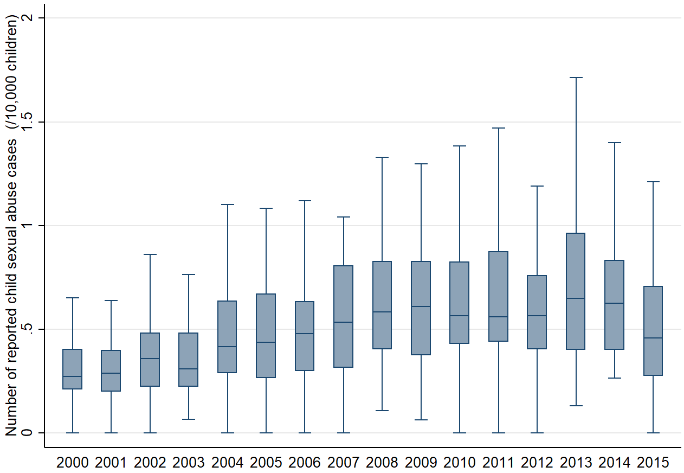

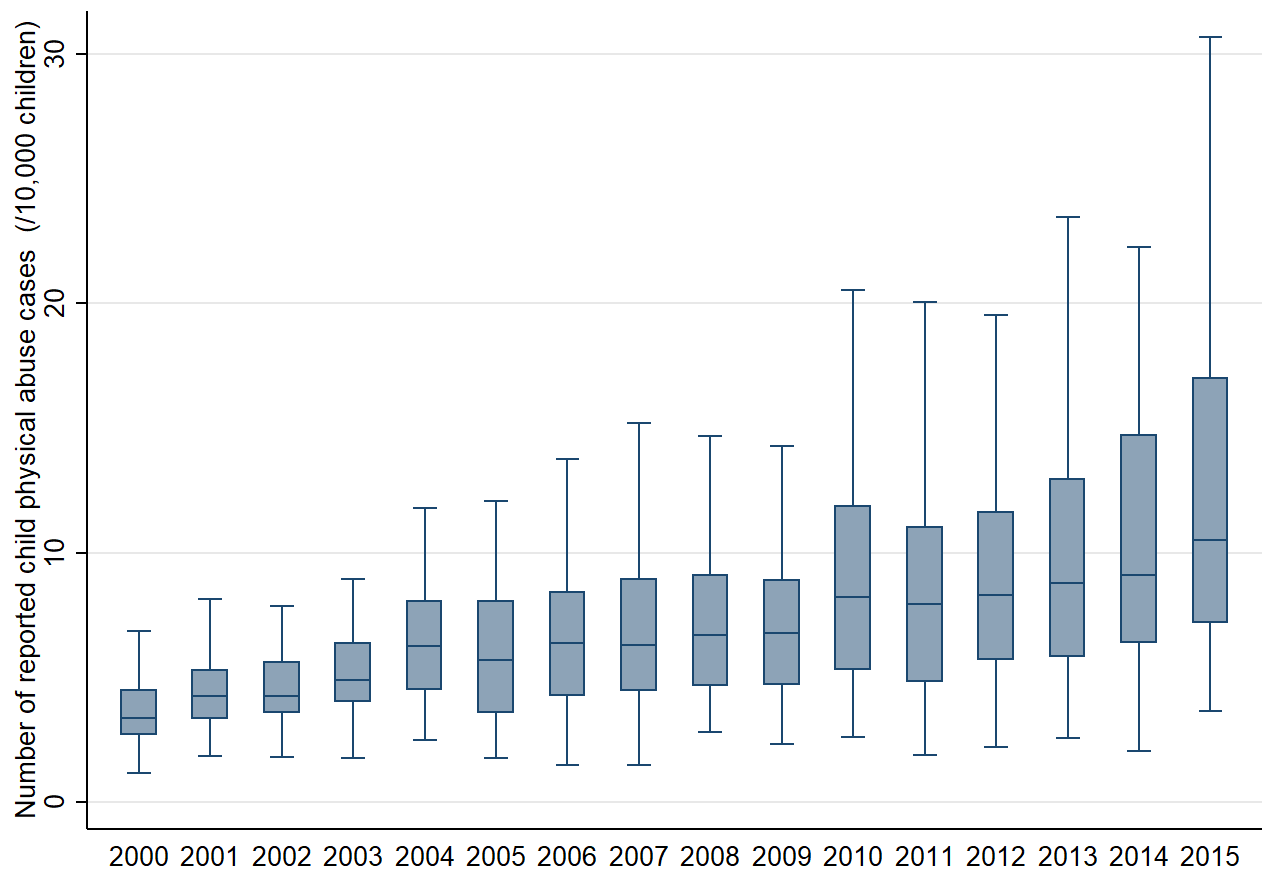


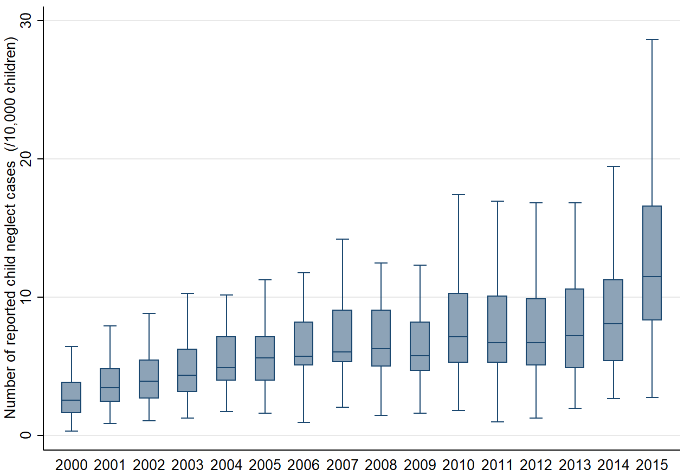

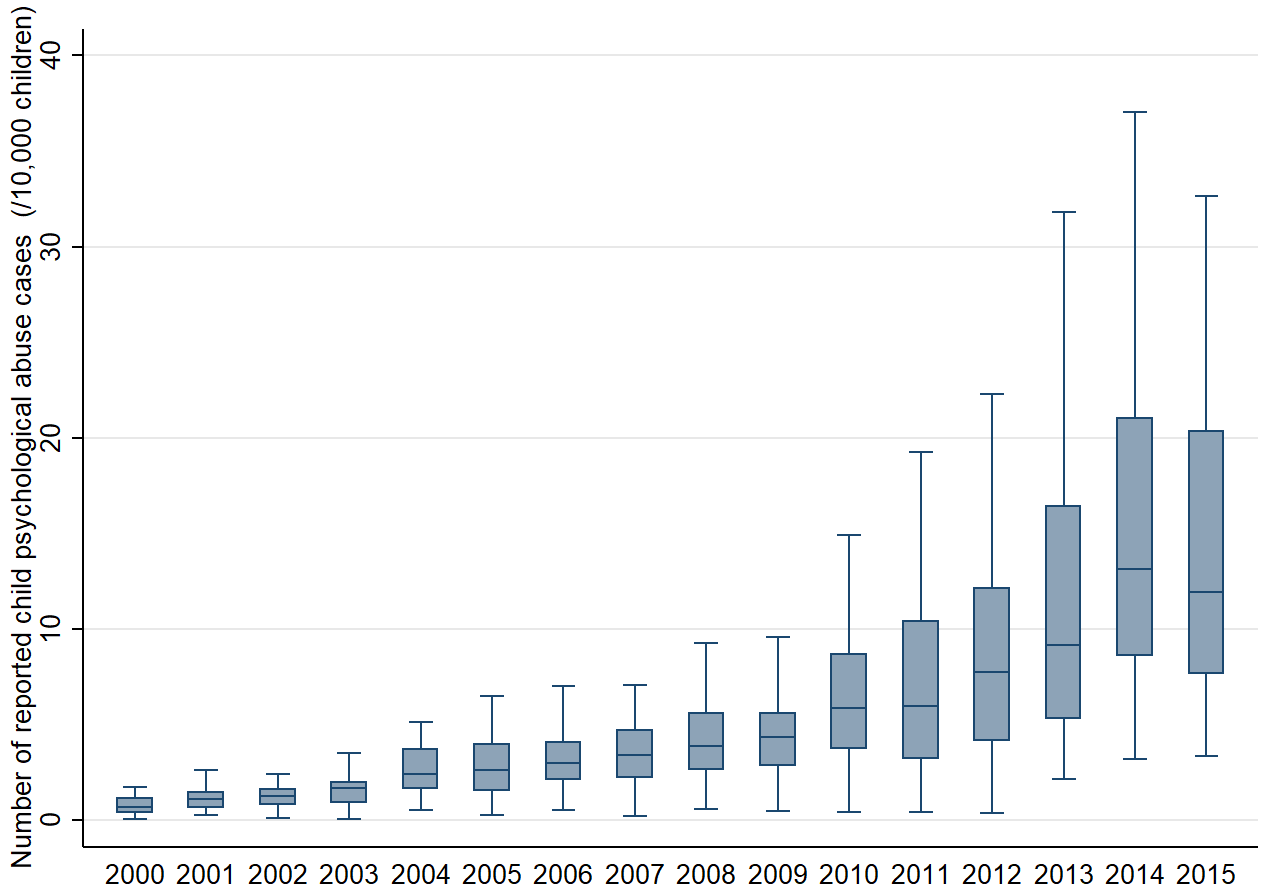


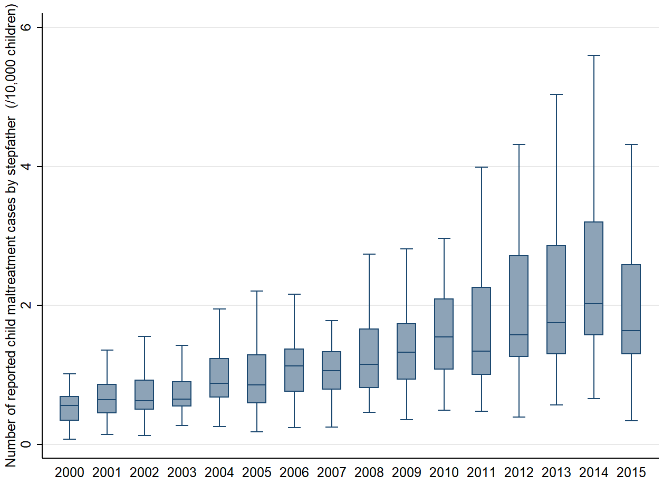

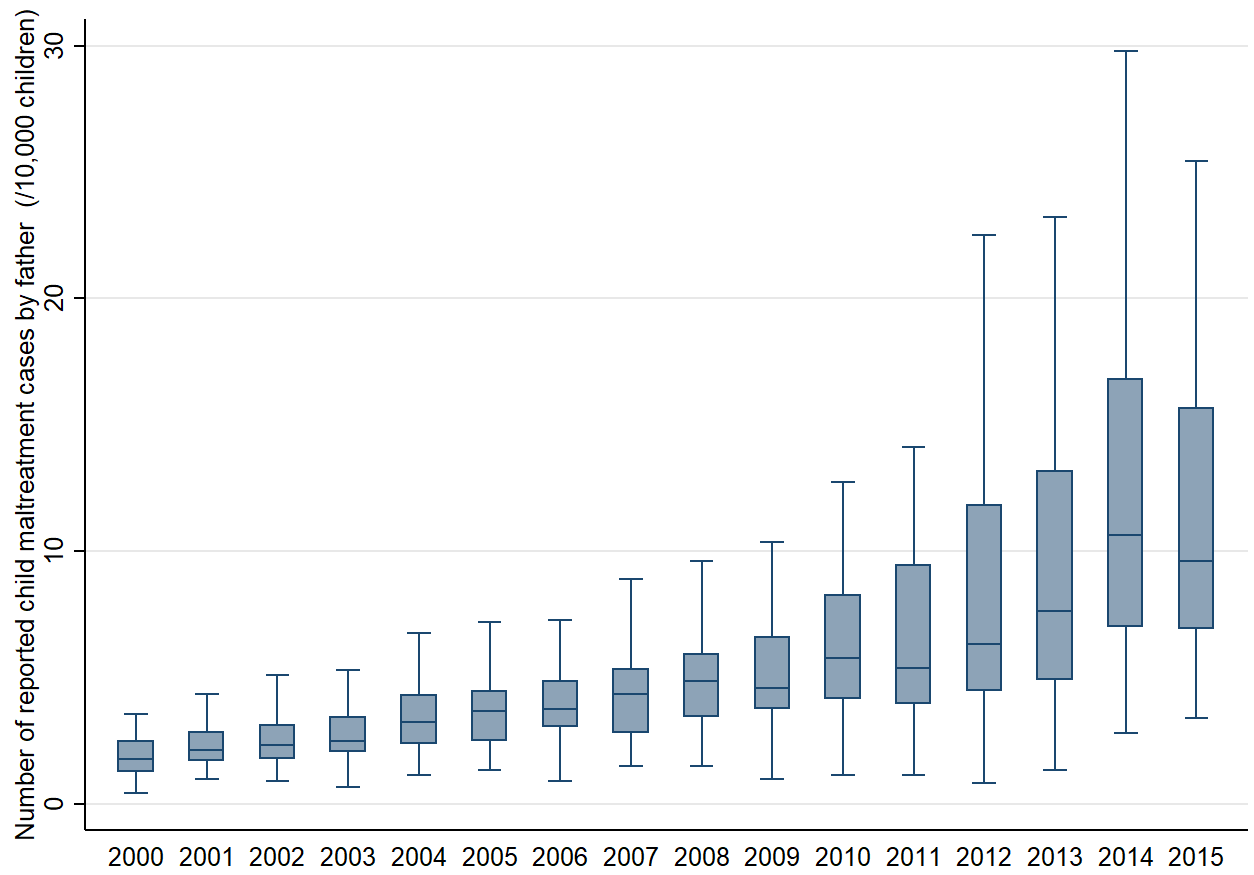


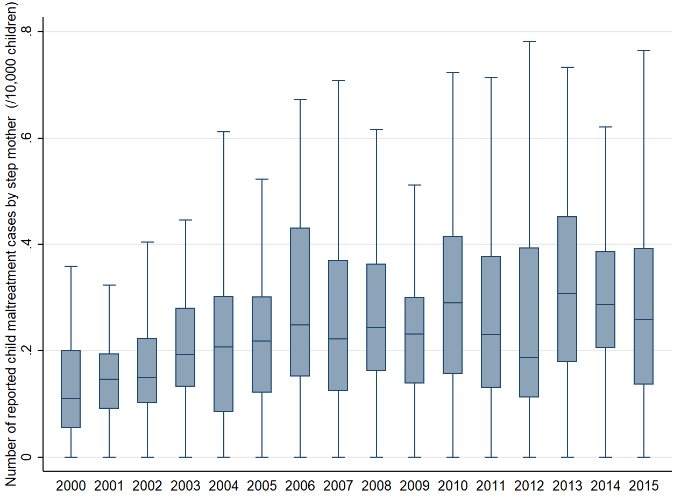

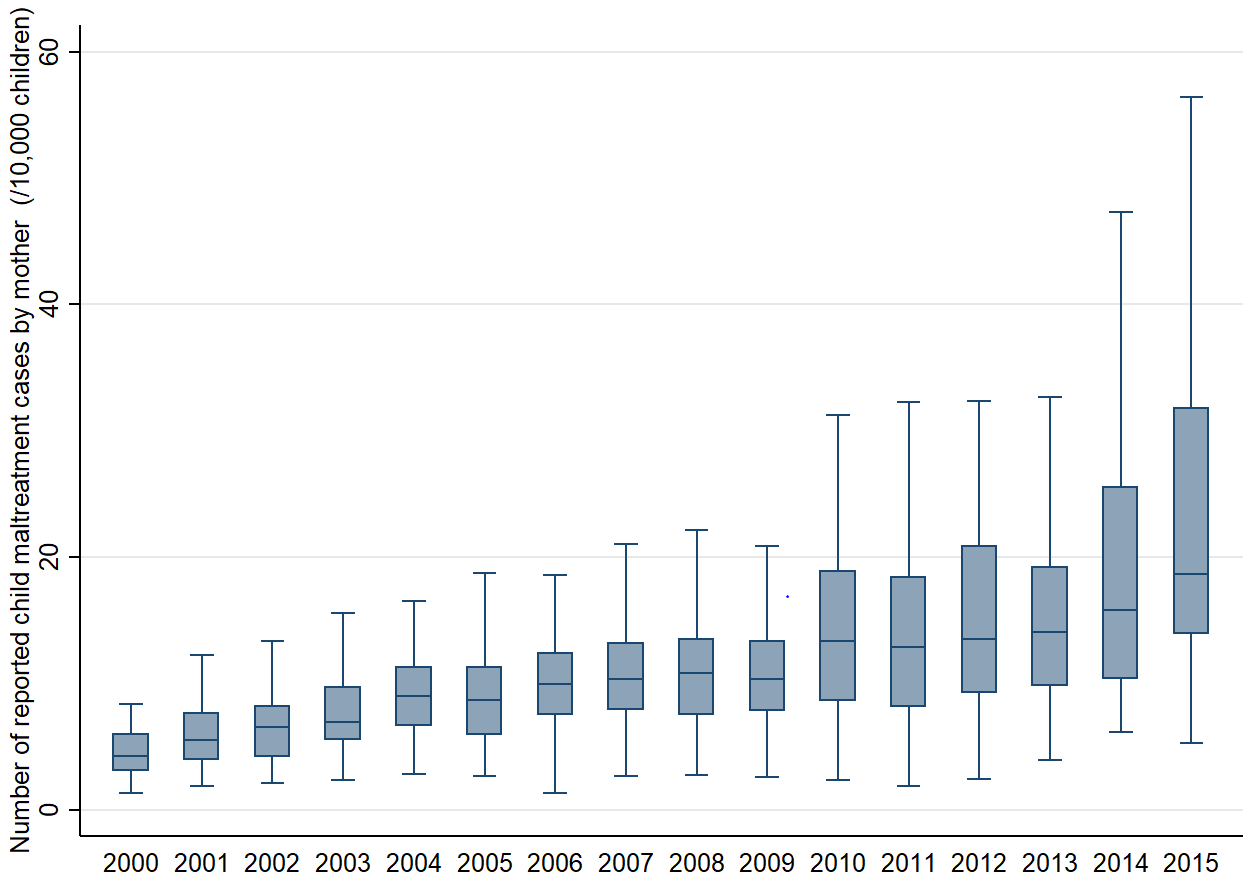

Supplement: Supplementary file 1 [file Data_Sheet_1.docx]
